# Paclitaxel Hypersensitivity: Is Titrated Dosing in Gynecologic Oncology Patients Necessary?

**DOI:** 10.3390/cancers18061018

**Published:** 2026-03-21

**Authors:** Ester Goldfeld, Leigh Cantrell, Marilyn Huang

**Affiliations:** 1 Department of Obstetrics and Gynecology, University of Virginia, Charlottesville, VA 22908, USA; eig5pe@uvahealth.org; 2 Division of Gynecologic Oncology, Department of Obstetrics and Gynecology, University of Virginia, Charlottesville, VA 22908, USA; lac6vz@uvahealth.org

**Keywords:** paclitaxel hypersensitivity, hypersensitivity reactions, chemotherapy

## Abstract

**Background/Objectives**: After several decades of using titrated paclitaxel infusions, our institution adopted non-titrated infusions in April 2023 to streamline infusion workflows. We aimed to evaluate whether this alteration in infusion was associated with a higher incidence of HSRs. **Methods**: This was a retrospective cohort study of patients receiving paclitaxel with titration versus non-titration from April 2022 through November 2023. Patients diagnosed with gynecologic cancers who presented for their first or second paclitaxel lifetime infusions were included. **Results**: A total of 150 patients were included in this study, each with one or two infusions, for a total of 282 infusion visits. There were 176 infusions performed with titrated paclitaxel (62.4%), and 106 infusions performed with non-titrated paclitaxel dose (37.6%). HSRs occurred in 20.8% of the non-titrated paclitaxel infusions and in 11.9% of titrated paclitaxel infusions (RR 1.73 (95% CI 1.006–3.006), *p* = 0.047). Additionally, when stratifying by first- or second-visit infusions, the HSR rate increased significantly for non-titrated infusions to 22.4% during the second visit, while there was a decrease to 8.4% for titrated infusions (RR 2.66 (95% CI 1.105–6.413), *p* = 0.029). Non-titrated infusion reactions were associated with higher grades of reaction. **Conclusions**: HSRs occurred more frequently with non-titrated infusions, particularly during the second administration, suggesting that eliminating titration may increase hypersensitivity risk. These findings support a prospective evaluation of titration rates to further refine paclitaxel administration.

## Simple Summary

Paclitaxel is one of the most widely used chemotherapeutic agents for treatment of gynecologic malignancies, but a standard titration and administration protocol does not exist. Our institution performed titrated dosing of the first two lifetime paclitaxel treatments due to concern for hypersensitivity reactions (HSRs), but transitioned to non-titrated dosing in April 2023. We observed an increase in HSR incidence associated with cessation of paclitaxel titration—specifically during the second lifetime administration—and grade of reactions were observed to be higher as well.

## 1. Introduction

Paclitaxel is widely used for the treatment of solid tumors and remains a cornerstone of therapy for gynecologic malignancies. Early studies attributed paclitaxel hypersensitivity reactions (HSRs) to polyoxyl 35 castor oil (Cremophor^®^ EL), a non-ionic emulsifier and solvent used to improve paclitaxel solubility [1,2,3]. Despite routine premedication with corticosteroids, diphenhydramine, and H_2_ antagonists, immediate HSRs occur in 10–40% of patients, with the majority occurring during the first or second lifetime exposure [4,5,6,7,8,9,10].

The exact mechanism of paclitaxel HSRs is not fully understood. Historically, reactions were thought to be primarily type I immunoglobulin E/G-mediated, resulting in symptoms such as facial flushing, pruritus, rash, abdominal pain, back pain, chest or throat tightness, and anaphylaxis. However, increasing evidence suggests that HSRs may also be due to non-IgE-mediated through complement activation-related pseudoallergy (CARPA) [11,12]. Cremophor EL can activate the complement cascade, generating C3a and C5a anaphylatoxins, which trigger mast cell and basophil activation and release of inflammatory mediators that contribute to HSRs [11,12]. This mechanism may explain why paclitaxel reactions frequently occur during the first exposure and often develop within minutes of starting the infusion. Experimental studies have shown that the Cremophor EL solvent used in conventional paclitaxel formulations can induce rapid complement activation in human serum and form micellar structures capable of triggering immune activation in vitro [9,10]. As complement activation occurs early during infusion and may be influenced by the initial concentration of Cremophor EL, infusion rate and priming technique may affect the risk of HSRs. Stepwise titration and differences in tubing priming may alter the amount of drug and solvent delivered at infusion initiation, potentially modifying early immune activation.

Common symptoms of paclitaxel HSRs include pruritus, hypotension, bronchospasms and dyspnea, and may progress to severe reactions with cardiovascular (CV) collapse [6,13]. Management requires immediate interruption of infusion and administration of supportive medications, including IV fluids, antipyretics, antihistamines, corticosteroids and epinephrine. For true suspected HSRs, consideration of a desensitization protocol or changing drug may be warranted.

Infusion practices vary widely among institutions, and current Food and Drug Administration (FDA) prescribing information does not provide guidance on titration protocols [10]. Some centers titrate the first one or two lifetime paclitaxel infusions, while others administer the drug at full rate from the start [12,13,14,15]. Historically, some institutions also primed intravenous tubing with drug (“hot priming”) to ensure accurate drug delivery, while others primed with diluent such as normal saline due to concerns regarding hazardous drug exposure, adoption of closed-system transfer devices, and improved pump accuracy [16]. Because priming and titration affect early Cremophor EL exposure, differences in these techniques may influence HSR risk.

Prior to April 2023, our institution utilized a titrated infusion protocol for the first and second lifetime paclitaxel infusions that included hot priming the IV line. In April 2023, this protocol was changed to non-titrated infusions without drug priming to improve efficiency and reduce chair time, based on reports suggesting acceptable safety without titration. Our primary aim was to compare the frequency of paclitaxel HSRs with titrated versus without non-titrated administration during the first two lifetime exposures. Secondary measures included whether HSR occurred in the first or second infusion, duration of infusion prior to HSR, and severity of reactions.

## 2. Materials and Methods

### 2.1. Study Design

After IRB exemption (IRB #24905), we identified patients aged 18 years and older who received their first or second lifetime paclitaxel infusion for the treatment of gynecologic malignancies, including ovarian, uterine, and cervical cancers. Data for titrated paclitaxel infusions was collected from April 2022 through March 2023. Non-titrated paclitaxel data was collected from April 2023 through November 2023. As the institutional protocol changed in April 2023, patients were managed according to the protocol in place at the time of their first paclitaxel infusion. Patients who received both first and second lifetime doses, thus, received either titrated infusions for both treatments or non-titrated for both treatments, and no patients received mixed protocols. Sociodemographic data included age, race/ethnicity, and gynecologic history. Clinical data were abstracted from the electronic medical record (EMR), including cancer diagnosis and history, prior treatments, paclitaxel titration status, dosing schedule (weekly vs. every 3 weeks), and occurrence of HSRs. Notably, HSRs were deemed to have occurred based on symptoms recorded in the EMR by staff administering the infusion and their clinical judgement at the time of symptom onset. If an HSR occurred, we then collected information regarding the grade of reaction, when the reaction occurred, supportive measures employed, and whether the paclitaxel infusion was restarted and completed.

### 2.2. Infusion Protocol

Our institutional paclitaxel titration protocol begins at 10 mL/h for 1 mL, followed by 20 mL/h for 1 mL, and then 30 mL/h for 1 mL for a total titration infusion time of approximately 11 min. After completion of the titration phase, the infusion was advanced to the full rate for the remainder of the dose. Non-titrated paclitaxel infusions were started at a predetermined rate based on the total volume to be infused over a three- or one-hour period using a fluid management pump. The same infusion protocol was used for both first and second lifetime doses for each patient. For titrated infusions, the IV tubing and in-line filter were primed with paclitaxel to ensure accurate dosing and eliminate air bubbles. In contrast, for non-titrated infusions, IV tubing was not primed with drug solution, and up to 20 mL of compatible fluid such as normal saline remained in the line at the start of the infusion. All patients received the same premedication regimen as per our institution guidelines, unless substitutions were warranted due to allergy or intolerance. Standard premedications included famotidine, dexamethasone, diphenhydramine, and antiemetics. When multiple agents were scheduled, paclitaxel was administered first.

### 2.3. Measures

The primary outcome was the frequency of HSRs in titrated versus non-titrated paclitaxel infusions. Secondary outcomes included whether HSRs occurred during the first or second infusion, duration of infusion at the time of reaction, and severity of HSR.

Based on events documented in the EMR by infusion center staff, the National Cancer Institute (NCI) Common Terminology Criteria for Adverse Events (CTCAE) version 5.0 was utilized to retrospectively assign the HSR as follows: Grade 1 is a mild transient reaction where infusion interruption is not indicated, or intervention is not indicated. For Grade 2 reactions, infusion interruption is indicated but responds promptly to symptomatic treatment or prophylactic medications are indicated for ≤24 h while Grade 3 is not rapidly responsive to symptomatic treatment and/or brief interruption of infusion, or symptoms recur following initial improvement or hospitalization is indicated for clinical sequelae. Grade 4 reactions are life-threatening, and urgent intervention is indicated, and Grade 5 is death.

### 2.4. Statistical Analysis

Baseline characteristics were analyzed at the patient level, whereas hypersensitivity outcomes were analyzed at the infusion level. Differences in HSR frequency between titrated and non-titrated paclitaxel infusions were analyzed using relative risk. Means with standard deviation were used to calculate averages, and Wilcoxon rank-sum tests were used for continuous variables. All statistical analyses were completed using SPSS (IBM SPSS Statistics for Mac, Version 29.0.2.0, Armonk, NY, USA: IBM Corp.) and GraphPad Prism (version 10.0.0 for Mac, GraphPad Software, Boston, MA, USA).

## 3. Results

The study included 150 unique patients who received their first and/or second lifetime paclitaxel infusion, for a total of 282 infusions analyzed. Ninety-three patients received titrated paclitaxel infusions, accounting for 176 infusions (93 first and 83 second lifetime infusions, respectively). In the non-titrated cohort, 57 patients received 106 infusions (57 first and 49 second lifetime infusions, respectively). Baseline characteristics were similar between the titrated and non-titrated cohorts, including a median age of 62 years, predominantly White patients, and primary diagnosis of ovarian or uterine cancer in most cases. Patient demographics presented in Table 1 are presented at the patient level, with each patient classified according to the infusion protocol used for both lifetime doses. No patients received combination of titrated and non-titrated infusions.

There were 5 infusions where paclitaxel was administered weekly over 1 h (1.8%), while the remaining 277 paclitaxel infusions (98.2%) were administered over 3 h every 3 weeks. Patients who received titrated paclitaxel had a significantly lower HSR rate (11.9%) compared to patients who received non-titrated infusion (20.8%), RR 1.73 (95% CI 1.006–3.006), *p* = 0.047 (Table 2). When further stratifying into first and second lifetime infusions, there was an increase in HSRs for patients receiving non-titrated paclitaxel during their second infusion (22.4% vs. 8.4%); RR 2.66 (95% CI 1.105–6.413), *p* = 0.029 (Table 2).

Common HSR symptoms recorded in the EMR included flushing, chest tightness, and back pain. All infusion reactions occurred during the 3 h paclitaxel infusions regardless of titration utilization. Most infusion reactions were Grade 1 or 2 (74.4%; Table 3). There was a higher percentage of Grade 2 HSRs in the titrated compared to the non-titrated group (85.7% vs. 59.1%), but this was not significant (*p* = 0.061). The titrated cohort had fewer Grade 3/4 reactions compared to the non-titrated cohort (9.6% vs. 31.8%, *p* = 0.088). Two Grade 4 HSRs occurred in patients receiving non-titrated paclitaxel. Both patients required hospitalization for further observation and permanently discontinued treatment with paclitaxel for the remainder of their cancer treatment. Approximately 70% of patients in both titrated and non-titrated cohorts were successfully rechallenged to complete paclitaxel infusion. Three patients, all in the non-titrated group, received only IV fluid intervention at the time of HSR; however, most patients in both groups received multiple interventions (81%). The timing of suspected HSR largely took place within the first 10 min of infusion in both cohorts (76.7%), *p* = 0.525. For titrated infusions, the average time to HSR from the start of the infusion was 7.9 min, and the amount of paclitaxel infused was approximately 4.28 mL; for non-titrated infusions, the average time to HSR from the start of the infusion was 10.7 min, and the amount of paclitaxel infused was approximately 36.6 mL.

Ten patients elected to discontinue treatment with paclitaxel after their first infusion for the titrated group, and eight patients discontinued treatment after their first infusion in the non-titrated group. Of the patients who had an HSR during their first infusion in the titrated group, there was only one patient with a recurrent HSR during the second infusion. In the non-titrated cohort, two patients who developed an HSR during their first infusion also experienced another reaction with their second infusion.

Patients who experienced an HSR were similar in age across both titration groups (Table 4). There was no difference in gynecologic cancer subtype or race/ethnicity between patients who experienced HSRs and those who did not.

## 4. Discussion

At our institution, titrated administration of paclitaxel was associated with a lower frequency of HSRs compared to non-titrated administration. Paclitaxel titration also resulted in a significantly lower rate of HSRs during the second infusion. These findings are consistent with prior reports demonstrating that titration protocols can reduce the incidence of HSRs. In our non-titrated cohort, patients experienced more Grade 3–4 HSRs, although they were equally to restart and complete treatment. Only 2.4% of HSRs in our cohort were Grade 1, which is consistent with some published studies [17] but lower than others (16.7%) [14].

We observed a longer interval from infusion start to symptom onset in the non-titrated cohort, corresponding with a greater volume of fluid infused before the reaction occurred. This differs from a prior study in which all infusion tubing was primed with paclitaxel; in that report, the non-titrated cohort experienced a shorter time to HSR compared to the titrated cohort (2 min vs. 17 min) [7]. The discrepancy in our findings is likely attributable to the presence of variable amounts of residual carrier fluid in the tubing of non-titrated infusions, as these lines were not primed. Priming the IV line for drugs such as rituximab rather than diluent has been associated with a reduced frequency of HSRs [18]; however, not priming tubing with paclitaxel and instead using saline can create an initial concentration gradient, and this practice may avoid the immediate Cremophor EL peak that triggers the complement cascade. Robust priming studies with paclitaxel have not been performed, but priming with paclitaxel may increase hazardous exposure risk for providers and patients and can prolong chair time during infusion setup.

An alternative approach to either titrating paclitaxel with drug-primed tubing or administering non-titrated infusions through diluent-primed lines is to use a titration protocol with the line primed using diluent. A recent abstract reported a significantly lower HSR rate when titration was performed through drug-primed tubing compared to non-titrated infusions with drug-primed lines (6% vs. 18.7%, *p* < 0.001). However, when titration was used, HSR rates did not differ significantly regardless of whether the line was primed with drug or diluent [19]. Accurate accounting of the priming volume is essential so that titration begins once paclitaxel reaches the patient. One study found that nurses who did not account for the initial 20 mL of saline in preprimed IV tubing had a higher incidence of HSRs than those who did. After the titration protocol was modified to include an initial 20 mL bolus to clear the priming volume, the paclitaxel HSR rate decreased to <1 in a 3-month period [20]. Alternatively, extending the titration duration (slower titration) may similarly mitigate HSRs by compensating for tubing fluid volume.

This study has several limitations. First, the retrospective, single-institution design introduces the potential for unrecognized confounding factors. Discontinuation of titration occurred concurrently with a change in tubing priming practice, making it difficult to determine the independent effects of these factors. As both titration and priming influence early exposure of Cremophor EL, either may have contributed to the observed difference in HSRs. In a prior study, standardized titration protocols demonstrated lower reaction rates [21]; however, these studies also did not isolate the effect of priming technique. Thus, our findings should be interpreted as reflecting the overall change in infusion protocol rather than the effect of titration alone.

Second, HSRs were identified based on documentation in the electronic medical record, and CTCAE grading was assigned retrospectively based on available documentation, introducing potential observer and data availability bias. Third, analyses were performed at the infusion level, and some patients contributed more than one observation. Because most patients contributed only one or two infusions and reactions typically occur during early exposures, infusions were treated as independent events, which does not account for correlation between infusions from the same patient. Next, the relatively small number of total HSRs limits the power to detect differences across subgroups or to evaluate risk factors such as comorbidities or premedication adjustments. However, the cohort sizes and number of HSRs experienced are also in line with the current literature. Finally, the non-overlapping time periods used to define the titrated and non-titrated cohorts may introduce temporal bias related to changes in infusion practices, staffing, or documentation over time. Despite these limitations, patient characteristics were similar between groups, and the observed differences in reaction rates are consistent with prior reports evaluating infusion protocols.

We observed a clear increase in HSR frequency following the institutional transition from titrated to non-titrated paclitaxel administration. Current high-level evidence to guide paclitaxel titration, with or without drug priming, remains limited. Importantly, recent data suggests that avoiding drug priming may be feasible if an optimized titration protocol is implemented [16]. Titration practices vary across institutions. For example, one institution primes the tubing with paclitaxel to the tip and administers 1 mL at 100 mL/h, pausing for 1 min and repeating this five times before infusing the full rate [22]. Another uses a stepwise protocol of 1% of the final rate for 15 min, then 10% for 15 min, followed by the full rate [14]. A third institution administers a 22 mL saline bolus at 660 mL/h for 2 min, then infuses paclitaxel sequentially at 10, 25, 50, and 100 mL/h for 5 min (20 min total) before transitioning to full rate [21]. Based on the observed increase in HSRs after discontinuing titration, our institution revised the paclitaxel administration protocol to reintroduce a brief stepwise infusion phase that accounts for saline priming. The updated protocol begins at 500 mL/h for 8 mL to clear the saline in the IV tubing, followed by 20 mL/h for 5 mL and 40 mL/h for 10 mL over 30 min, before advancing to the full infusion rate for the remainder of the dose. We anticipate that these modifications will better mitigate HSR risk among patients receiving first and second lifetime paclitaxel.

## 5. Conclusions

Reducing HSRs has the potential to minimize treatment interruptions, decrease patient anxiety, and reduce healthcare costs associated with additional interventions. Robust studies within gynecologic cancers alone have historically not been performed, as gynecologic cancers are commonly grouped breast and lung cancers [23]. Given the observed lower incidence of HSRs with titrated paclitaxel infusions for patients with gynecologic malignancies at our institution, the next step is to prospectively evaluate titration rates and diluent priming to further refine paclitaxel administration. First, we believe that it is more prudent to prospectively evaluate various titration rates and their impact on HSRs, taking into account our own institution’s revised protocol and comparing to a longer titration protocol without drug-priming but, rather, careful saline priming given previously described data on minimal effects of drug priming on HSR incidence if titration is performed.

## Figures and Tables

**Table 1:** Patient level baseline characteristics according to titrated versus non-titrated paclitaxel protocol.

Characteristic	Overall (*n* = 150)	Titrated (*n* = 93)	Non-Titrated (*n* = 57)
Age (Median)	62	65	59
Race/Ethnicity			
White	118 (78.6%)	72 (77.4%)	46 (80.7%)
Black	21 (14.0%)	14 (15.1%)	7 (12.2%)
Latino	7 (4.6%)	4 (4.3%)	3 (5.2%)
Asian/Pacific Islander	3 (2.0%)	2 (2.2%)	1 (1.9%)
Other/Multiracial	1 (0.8%)	1 (1.0%)	0 (0%)
GYN Cancer			
Uterine	70 (46.6%)	45 (48.4%)	25 (43.8%)
Ovarian/Fallopian Tube/Peritoneal	72 (48.0%)	43 (46.2%)	29 (50.9%)
Cervical	8 (5.4%)	5 (5.4%)	3 (5.3%)

**Table 2:** Infusion-level HSRs according to titrated versus non-titrated paclitaxel administration. Each paclitaxel administration was analyzed as a separate event; patients receiving both first and second lifetime doses contributed to two infusion events.

	Titrated	Non-Titrated	Relative Risk [95% CI]	*p*
Total Infusion (*n*)	176	106	1.73	
Frequency of HSR, *n* (%)	21 (11.9%)	22 (20.8%)	[1.006–3.006]	0.047
First Lifetime Infusion (*n*)	93	57	1.28	
Frequency of HSR, *n* (%)	14 (15.1%)	11 (19.2%)	[0.625–2.627]	0.498
Second Lifetime Infusion (*n*)	83	49	2.66	
Frequency of HSR, *n* (%)	7 (8.4%)	11 (22.4%)	[1.105–6.413]	0.029

**Table 3:** HSR characteristics. CTCAE grade of HSRs was not statistically different amongst titrated and non-titrated cohorts. Most patients were able to finish their infusion following pause to address HSR, and the majority of patients received multiple interventions once an HSR was noted. The time to infusion reaction from the start of an infusion for most HSRs was within the first 10 min for both titrated and non-titrated HSRs, with few HSRs occurring 11–30 min following the start of a paclitaxel infusion.

	Overall (43 HSR)	Titrated (21)	Non-Titrated (22)	*p*
CTCAE Grade				
1	1 (2.4%)	1 (4.7%)	0 (0%)	0.488
2	31 (72.1%)	18 (85.7%)	13 (59.1%)	0.061
3	9 (20.9%)	2 (9.6%)	7 (31.8%)	0.088
4	2 (4.6%)	0 (0%)	2 (9.1%)	0.294
Able to Finish				0.816
No	13 (30.3%)	6 (28.6%)	7 (31.8%)	
Yes	30 (69.7%)	15 (71.4%)	15 (68.2%)	
Interventions				0.850
Antihistamine	1 (2.4%)	1 (4.8%)	0 (0%)	
IV Fluids	3 (6.9%)	0 (0%)	3 (13.6%)	
Famotidine	1 (2.4%)	0 (0%)	1 (4.4%)	
Steroids	3 (6.9%)	2 (9.5%)	1 (4.4%)	
Multiple Interventions	35 (81.4%)	18 (85.7%)	17 (77.3%)	
Time to HSR				
1–10 min	33 (76.7%)	17 (80.9%)	16 (72.7%)	0.525
11–30 min	7 (16.2%)	3 (14.2%)	4 (18.1%)	0729
>30 min	2 (4.6%)	1 (4.9%)	1 (4.6%)	0.973
After Completion	1 (2.5%)	0 (0%)	1 (4.6%)	0.491

**Table 4:** Patient HSR factors. There was no significant difference in patient factors such as age and race/ethnicity in those that developed HSRs when compared to those that did not. Additionally, the type of gynecologic cancer did not influence whether or not HSR occurred.

Characteristic	HSR Present (*n* = 40)	HSR Absent (*n* = 110)	*p*
Age (Median)	63	62	0.292
Race/Ethnicity			0.541
White	30 (75.0%)	88 (80.0%)	
Black	6 (15.0%)	15 (13.6%)	
Latino	2 (5.0%)	5 (4.6%)	
Asian/Pacific Islander	2 (5.0%)	1 (1.4%)	
Other/Multiracial	0 (0%)	1 (1.4%)	
GYN Cancer			0.381
Uterine	15 (37.5%)	55 (50.0%)	
Ovarian/Fallopian Tube/Peritoneal	22 (55.5%)	50 (45.5%)	
Cervical	3 (7.5%)	5 (4.5%)	

## Data Availability

The data presented in this study are available on request from the corresponding author.

## Abbreviations

The following abbreviations are used in this manuscript:

HSRs	Hypersensitivity reactions
CV	Cardiovascular collapse
FDA	Food and Drug Administration
EMR	Electronic medical record
CTCAE	Common Terminology Criteria for Adverse Events
NCI	National Cancer Institute
RR	Relative risk
CI	Confidence interval
